# THE GROWING EVIDENCE OF THE RELATIONSHIP BETWEEN OBESITY AND CANCER AND THE ROLE OF BARIATRIC SURGERY

**DOI:** 10.1590/0102-6720202400044e1838

**Published:** 2024-12-02

**Authors:** Paulo KASSAB, Álvaro Antônio Bandeira FERRAZ, Anna Clara Hebling MITIDIERI, Luiz Vicente BERTI, Marco Aurélio SANTO, Tiago SZEGO, Caio de Carvalho ZANON, Osvaldo Antônio Prado CASTRO, Wilson Rodrigues de FREITAS, Elias Jirjoss ILIAS, Carlos Alberto MALHEIROS, Antônio Carlos VALEZ, Antônio Carlos Ligocki CAMPOS, Brazilian College of Digestive Surgery (CBCD) and Brazilian Society of Bariatric and Metabolic Surgery (SBCBM).

**Affiliations:** 1 Santa Casa de São Paulo, Faculdade de Ciências Médicas, Department of Surgery – São Paulo (SP), Brazil; 2 Universidade Federal de Pernambuco, Hospital Universitêrio, Department of Surgery – Recife (PE), Brazil; 3 Santa Casa de Misericórdia de São Paulo, Department of Surgery – São Paulo (SP), Brazil; 4 Universidade de São Paulo, Faculty of Medicine, Gastroenterology Department – São Paulo (SP), Brazil; 5 Santa Casa de São Paulo, Faculdade de Ciências Médicas – São Paulo (SP), Brazil; 6 Universidade Estadual de Londrina, Department of Surgery, Digestive System Surgery – Londrina (PR), Brazil; 7 Universidade Federal do Paranê, Faculdade de Medicina de Curitiba, Department of Surgery – Curitiba (PR), Brazil

**Keywords:** Obesity, Bariatric surgery, Neoplasms, Obesidade, Cirurgia bariêtrica, Neoplasias

## Abstract

Obesity is recognized as a significant risk factor for various types of cancer. Although the incidence of some types of cancer across various primary sites is decreasing due to specific prevention measures (screening programs, smoking cessation), the incidence of neoplasms in the young population shows a significant increase associated with obesity. There is sufficient evidence to say that bariatric surgery has been shown to significantly lower the risk of developing obesity-associated cancers, which are linked to metabolic dysregulation, chronic low-grade systemic inflammation, and hormonal alterations such as elevated levels of insulin and sex hormones.

## RESUMO

A obesidade é reconhecida como um fator de risco significativo para vêrios tipos de câncer. Embora a incidência de alguns tipos de câncer de vêrios locais primêrios esteja diminuindo devido a medidas específicas de prevenção (programas de triagem, cessação do tabagismo), a incidência de neoplasias na população jovem mostra um aumento significativo associado à obesidade. Hê evidências suficientes para dizer que a cirurgia bariêtrica demonstrou reduzir significativamente o risco de desenvolver cânceres associados à obesidade, que estão ligados à desregulação metabólica, inflamação sistêmica crônica de baixo grau e alterações hormonais, como níveis elevados de insulina e hormônios sexuais.

## SUMMARY OF THE MAIN RECOMMENDATIONS

• Obesity is recognized as a significant risk factor for various types of cancer.

• Although the incidence of some types of cancer at different primary sites is decreasing due to specific prevention measures (screening programs, smoking cessation), the incidence of neoplasms in the young population shows a significant increase associated with obesity.

• Bariatric surgery has been shown to significantly lower the risk of developing obesity-associated cancers, which are linked to metabolic dysregulation, chronic low-grade systemic inflammation, and hormonal alterations such as elevated insulin and sex hormone levels.

## INTRODUCTION

The worldwide increase in obesity has been clearly observed. Kaidar-Person et al., back in 2011, stated that the 21^st^ century was dealing with 2 major epidemics: obesity and cancer^33^. The prevalence of obesity in the United States (U.S.) increased from 30.5 to 41.9% between 2000 and 2020, while the prevalence of severe obesity increased from 4.7 to 9.2% during the same period^64^. Another study found that the incidence of obesity increased by 18% between 2013 and 2017 compared to 2009 to 2013^42^. Obesity-related conditions, including heart disease, stroke, type 2 diabetes, and certain types of cancer, are among the leading causes of premature death and are preventable^12^.

Additionally, projections published in 2019 suggest that by 2030, nearly 1 in 2 adults in the U.S. will have obesity, with severe obesity becoming increasingly prevalent^69,72^. In the young population, the 2023 guidelines from the American Academy of Pediatrics reported that the percentage of U.S. children and adolescents affected by obesity has more than tripled, rising from 5% in 1963–1965 to 19% in 2017–2018^28,69^.

In Brazil, evidence indicates a significant increase in the prevalence of obesity over recent decades. A systematic review and meta-analysis by Kodaira et al. found that the pooled prevalence of obesity in Brazilian adults increased by 15% from 1974–1990 to 2011–2020. This trend was observed in both men and women across almost all periods^34^. Similarly, Estivaleti et al. reported that the prevalence of obesity in Brazilian adults rose from 11.8% in 2006 to 20.3% in 2019, with projections suggesting that obesity may affect 29.6% of adults by 2030.This study also highlighted that women, black and other minority ethnicities, and individuals with lower educational attainment are particularly at risk^24^.

Several medical conditions are associated with obesity, including heart disease, hypertension, stroke, type 2 diabetes, metabolic syndrome, and certain cancers^71^. These are among the leading causes of preventable premature death. The estimated annual medical cost of obesity in the U.S. was nearly $173 billion (in 2019 dollars), with medical costs for obese adults being $1,861 higher than for people at a healthy weight^60^.Other studies have shown that the cost of inpatient surgical procedures, both oncologic and benign, abdominal and non-abdominal, is higher in obese patients^56^.

### Evidence of the relationship between obesity and cancer

Obesity is recognized as a significant risk factor for various types of cancer. According to the American Cancer Society, excess body fat contributes substantially to cancer risk, with approximately 10.9% of cancer cases in women and 4.8% in men in the U.S. attributed to overweight or obesity^61^. In the United Kingdom (UK), overweight and obesity were the second most common preventable causes of cancer, accounting for 6.3% of all cases in 2015. The proportion was higher in women (7.5%) than in men (5.2%), with the highest rates observed in Scotland (6.8%) and the lowest in Wales (5.4%). The type of cancer most strongly associated with overweight and obesity in women was uterine cancer (34.0%), while in men, it was esophageal cancer (31.3%)^9^.

The cause-and-effect relationship between obesity and cancer seems to be even more important^8^. Although the incidence of certain types of cancer at various primary sites is decreasing due to specific prevention measures (*e.g*., screening programs, smoking cessation), the incidence of neoplasms in younger populations has shown a significant increase associated with obesity.

A large British population-based study involving 5.24 million individuals with 166,955 new cases across 22 types of cancer noted a relationship between body mass index (BMI) and certain types of cancer. Of the more than 5 million individuals, 166,955 developed cancers. BMI was associated with 17 of the 22 types of cancer, though the effects varied substantially by site. Each 5 kg/m^2^ increase in BMI was linearly associated with the incidence of uterine, cervical, thyroid, and leukemia cancers. High BMI was also associated with liver, colon, ovarian, and postmenopausal breast cancer in general (all p<0.0001), with these effects varying according to individual characteristics^22,36^. It is estimated that 41% of uterine cancers and 10% or more of gallbladder, kidney, liver, and colon cancers could be attributed to excess weight. A 1 kg/m^2^ increase in BMI across the population could result in 3,790 additional cancer cases per year in the UK^7^.

A 2015 study reported the risk of cancer attributable to obesity by gender: in women, around 30% for breast and uterine cancers, around 10% for colon, rectum, and gallbladder cancers, 8.8% for kidney cancer, 3.4% for pancreas cancer, and 1.2% for esophageal cancer. In men, obesity was responsible for over 50% of colon and rectal cancers, 24.8% of kidney cancers, and around 10% for pancreatic and esophageal cancers^4^.

In addition to these types of cancer, other neoplasms associated with overweight and obesity include meningiomas, thyroid cancer, multiple myeloma, ovarian cancer, liver cancer, and esophagogastric junction cancer. From 1995 to 2014, the incidence of obesity-related neoplasms among adults aged 25–49 years increased significantly in 6 types of cancers: multiple myeloma, colorectal, uterine, gallbladder, kidney, and pancreatic cancer. This association was more evident in women, as described in a 17-year study (January 2000 to December 2016), which reported a shift in the age distribution of obesity-associated cancers, with 70.3% of patients being female. In fact, a BMI greater than or equal to 30 corresponds to an 86% increase in the relative risk of colorectal cancer in young women, with the risk increasing by 18% for every 5-unit rise in BMI, even in women with no family history of this type of cancer^41^.

In an interesting review article, Pati et al. focused on the epidemiology and relationship between obesity and cancer. They concluded that around 4 to 8% of all cancers can be attributed to obesity, which is now recognized as a risk factor for several types of cancer, including postmenopausal breast, liver, gallbladder, pancreas, colon and rectum, endometrial, kidney, and esophageal cancer. They also noted that excess body fat resulted in an increased risk of approximately 17% in cancer-specific mortality^46^.

A large cohort study in Spain, involving more than 2.5 million individuals from 2009 to 2018, analyzed individuals over 45 years of age who were cancer-free in 2009. After 9 years of follow-up, 225,396 participants were diagnosed with cancer. This analysis demonstrated that longer duration and higher degrees of obesity, particularly in younger individuals and those who became overweight or obese during early adulthood, increased the risk of 18 types of cancer, including leukemia and non-Hodgkin lymphoma. Among never-smokers, head and neck and bladder cancers, not yet recognized as obesity-related cancers in the literature, were also linked to obesity. The authors emphasized the importance of including obesity prevention strategies in public health programs^49^.

In summary, according to the literature, increased body weight and obesity are related to at least 13 types of cancer, 6 of which are located in the digestive system (Table 1).

**Table 1:** Evidence of the relationship between high body mass index and types of cancer^37^.

Strength of evidence for a cancer-preventive effect of the absence of excess body fat according to the site or type of cancer		
Location or type of cancer	Strength of evidence in humans	Relative risk of higher versus normal BMI (95%CI)
Esophagus adenocarcinoma	Sufficient	4.8 (3.0-7.7)
Cardia	Sufficient	1.8 (1.3-2.5)
Colorectal	Sufficient	1.3 (1.3-1.4)
Liver	Sufficient	1.8 (1.6-2.1)
Gallbladder	Sufficient	1.3 (1.2-1.4)
Pancreas	Sufficient	1.5 (1.2-1.8)
Breast, post-menopausal	Sufficient	1.1 (1.1-1.2)
Uterus (body)	Sufficient	7.1 (6.3-8.1)
Ovary	Sufficient	1.1 (1.1-1.2)
Kidney (renal cell)	Sufficient	1.8 (1.7-1.9)
Meningioma	Sufficient	1.5 (1.3-1.8)
Thyroid	Sufficient	1.1 (1.0-1.1)
Myeloma	Sufficient	1.5 (1.2-2.0)
Male Breast Cancer	Limited	Not applicable
Prostate Cancer (fatal)	Limited	Not applicable
Diffuse Large B-cell Lymphoma	Limited	Not applicable

BMI: body mass index; 95%CI: 95% confidence interval. A five-point increase in BMI has an impact on the relative risk (RR) of several cancers associated with excess weight, including cervical cancer (worldwide RR – 1.5), esophageal cancer (RR in Europe and North America – 1.48), liver cancer (European RR – 1.59), pancreatic cancer (RR in Europe, Australia, and North America – 1.14), and stomach cancer (RR in Europe and North America – 1.31)^67^. Additionally, evidence suggests that obesity exacerbates several aspects related to cancer survival, including quality of life, recurrence, progression, and prognosis, while also increasing the risk of a second primary neoplasm. During cancer treatment itself, obesity is related to complications such as lymphedema in breast cancer and incontinence in prostate cancer following radical surgical resection. Even in hematologic malignancies, such as multiple myeloma, patients with higher BMI face a higher risk of death^31^.

The relationship between BMI and the risk of developing and dying from cancer is so direct that it is estimated that, in a hypothetical situation where the American population did not exceed a BMI of 25 kg/m^2^, 90,000 fewer cancer-related deaths would occur each year in the U.S.^7,11,70^.

The mechanisms linking obesity to cancer are multifactorial. Obesity is associated with metabolic dysregulation, chronic low-grade systemic inflammation^27^, and hormonal changes, including elevated insulin and sex hormones, which can promote carcinogenesis. Increased circulating estrogens, tumor cell growth and migration, modification of the tumor microenvironment, and neoangiogenesis have also been implicated. Obesity induces a variety of systemic changes, including altered levels of insulin, insulin-like growth factor-1 (IGF-1), leptin, adiponectin, steroid hormones, and cytokines, creating an environment that favors tumor initiation and progression. Chronic low-grade inflammation, driven by adipose tissue dysfunction and macrophage infiltration, is a hallmark of obesity and contributes to both cancer risk and progression. Adipose tissue in obese individuals secretes a large number of biologically active substances termed adipokines, which include pro-inflammatory cytokines like IL-6 and TNF-α. These adipokines can induce pathological alterations in insulin pathways and promote a pro-inflammatory state, both of which are linked to increased cancer risk. Additionally, obesity-related changes in the gut microbiome and adipokine pathways further contribute to cancer risk^30,32,59,62^.

One hypothesis is that adipose tissue acts as an organ capable of releasing enzymes and other chemical mediators, such as estrogens, whose increase results from heightened aromatase activity in adipose tissue^50^. Obesity also impacts the tumor microenvironment by modulating immune cell infiltration, influencing cancer progression and response to therapy^15^.

Therefore, the pathophysiology of the relationship between obesity and cancer is quite complex and not yet fully understood, as it involves two multifactorial conditions encompassing genetic, environmental, and social factors in a highly interconnected manner. However, several obesity-related changes have been shown to influence carcinogenesis, including hyperinsulinemia, elevated leptin levels, chronic inflammation, oxidative stress, HIF-1α activation, cytokine secretion, DNA methylation, visceral adipose dysfunction, release of adiponectin, exosome miRNA release, and changes in the metabolism of sex hormones^74^.

As for weight loss, studies have shown a reduced risk of breast, endometrial, colorectal, and prostate cancers in patients who lost weight. A large prospective study with postmenopausal women showed that intentional loss of more than 5% of body weight was associated with a lower risk of obesity-related neoplasms, though this effect was not observed when weight loss was unintentional^39,65^. Lifestyle modifications could prevent a substantial proportion of cancer cases. A study found that adherence to a healthy lifestyle — defined as non-smoking, moderate alcohol consumption, maintaining a BMI between 18.5 and 27.5, and engaging in regular physical activity — could prevent 25% of cancer cases in women and 33% in men^9,16,51,63^.

Obesity negatively impacts all phases of cancer treatment, increasing the incidence of at least 13 types of cancer and delaying diagnosis due to technical difficulties in the surgical treatment, difficulties in accessing diagnostic methods, or the stigma that obese patients face, which may discourage them from seeking medical attention. Obesity also increases therapeutic complications (surgeries, radiotherapy, chemotherapy, etc.) and adversely affects survival rates in cancer patients. It compromises quality of life, increases the likelihood of cancer recurrence and progression, and raises the risk of developing new neoplasms^10,11,25,57,66^.

In conclusion, there seems to be sufficient evidence to link obesity to cancer. The question that follows is: does bariatric surgery reduce cancer risk?

### Does Bariatric Surgery reduce cancer risk?

Obesity surgery reduces the incidence of cancer. Bariatric surgery has been shown to significantly lower the risk of developing obesity-associated cancers, which are linked to metabolic dysregulation, chronic low-grade systemic inflammation, and hormonal alterations such as elevated insulin and sex hormone levels.

A systematic review and meta-analysis demonstrated that bariatric surgery is associated with a reduced overall incidence of cancer (RR 0.62, 95%CI 0.46–0.84) and obesity-related cancer (RR 0.59, 95%CI 0.39–0.90). Another study found that bariatric surgery was associated with a significantly lower incidence of obesity-associated cancer (adjusted HR 0.68, 95%CI 0.53–0.87) and cancer-related mortality (adjusted HR 0.52, 95%CI 0.31–0.88)^74^.

Additionally, a multi-center population-based study reported that the cumulative incidence of cancers among patients who experienced obesity recurrence was significantly lower in the bariatric surgery group compared to the nonsurgical control group (HR 0.482, 95%CI 0.459–0.507). This protective effect extends to specific cancers such as breast, endometrial, and colorectal cancers^2,13^.

Other studies have shown that surgical treatment of obesity is associated with reduced risks of neoplasms in general, hormone-dependent cancers (breast, endometrium, and prostate), and obesity-related cancers (postmenopausal breast, endometrium, and colorectal)^40,54,68^.

In summary, bariatric surgery not only aids in weight loss but also significantly reduces the risk of developing various obesity-associated cancers by mitigating the underlying metabolic and inflammatory pathways that promote carcinogenesis. The benefits attributed to bariatric surgery in reducing cancer incidence also extend to other aspects of cancer treatment, including improved outcomes in adjuvant treatments (chemo and radiotherapy), lower recurrence rates, and increased overall survival. Therefore, there is substantial evidence that bariatric surgery positively affects the key pathophysiological mechanisms linking obesity and cancer^12^. The mechanisms include reduction in insulin and leptin levels, chronic inflammation, and oxidative stress, as well as the restoration of sex hormone levels, especially estradiol. Additionally, bariatric surgery has a significant impact on the intestinal microbiome and serum proteomics^60^.

In 2004, a study involving 1,035 patients who underwent bariatric surgery, compared with a cohort of more than 5,000 non-operated patients, demonstrated a significant decrease in cancer cases. In those who underwent surgery, the incidence was 2.03%, while in the non-surgical group, it was 8.49%, indicating that bariatric surgery reduced cancer incidence fourfold. There was also a large decrease in the number of hospitalizations for cancer: 54.95/1,000 person-years in the non-operated group *versus* only 11.80 in the operated group^16^. In the same year, an epidemiological evaluation of 15,850 patients, 7,925 of whom underwent surgery compared to an equal number of controls, showed a 60% decrease in cancer mortality^11^.

A few years later, a prospective, controlled analysis carried out in Sweden, involving more than 4,000 patients, about half of whom underwent bariatric surgery compared to controls, revealed the occurrence of 47 cases of cancer in the non-operated group compared to 29 in the operated group^61^. In 2017, another observational study with the impressive number of 18,355 operated patients, compared to more than 40,000 non-operated controls, revealed a significant decrease in cancer mortality, with greater weight loss associated with lower mortality^55^.

A recent systematic review and meta-analysis of 32 studies involving patients with obesity who underwent bariatric surgery compared to controls managed with conventional treatment analysis suggested that bariatric surgery was associated with a reduced overall incidence of cancer (RR 0.62, 95%CI 0.46–0.84, p<0.002), obesity-related cancer (RR 0.59, 95%CI 0.39–0.90, p=0.01), and cancer-associated mortality (RR 0.51, 95%CI 0.42–0.62, p<0.00001). For specific cancers, bariatric surgery was associated with a reduction in the future incidence of hepatocellular carcinoma (RR 0.35, 95%CI 0.22–0.55, p<0.00001), colorectal cancer (RR 0.63, 95%CI 0.50–0.81, p=0.0002), pancreatic cancer (RR 0.52, 95%CI 0.29–0.93, p=0.03), and gallbladder cancer (RR 0.41, 95%CI 0.18–0.96, p=0.04), as well as female-specific cancers, including breast cancer (RR 0.56, 95%CI 0.44–0.71, p<0.00001), endometrial cancer (RR 0.38, 95%CI 0.26–0.55, p<0.00001), and ovarian cancer (RR 0.45, 95%CI 0.31–0.64, p<0.0001). There was no significant reduction in the incidence of esophageal, gastric, thyroid, kidney, prostate cancer or multiple myeloma after bariatric surgery as compared to patients with morbid obesity who did not have bariatric surgery. The authors concluded that bariatric surgery might decrease future overall cancer incidence and mortality, particularly in relation to seven obesity-related cancers^74^.

Regarding cancers of the upper digestive tract, with the exception of colon cancer, hundreds of thousands of bariatric surgeries have been performed to date. However, a systematic review analyzing the relationship between bariatric surgeries and the reduction of digestive cancer specifically revealed that only a few cases of cancer after the operations were described. Therefore, there is no conclusive evidence supporting a correlation between obesity surgery and upper gastrointestinal cancer^23^. Åkerström et al. evaluated 748,932 participants diagnosed with obesity, of whom 91,731 underwent bariatric surgery, predominantly gastric bypass (n=70,176; 76.5%). The adjusted risk of esophageal cancer decreased over time after gastric bypass, from 2.2 (95%CI 0.9–4.3) after 2 to 5 years to 0.6 (95%CI <0.1–3.6) after 10 to 40 years. Gastric bypass patients also had a reduced risk of adenocarcinoma of the cardia compared with non-operated patients with obesity (adjusted HR 0.6, 95%CI 0.4–1.0 (0.98)), with point estimates decreasing over time. Gastric bypass was followed by a strongly decreased adjusted risk of esophageal adenocarcinoma (HR 0.3, 95%CI 0.1–0.8) but not of cardia adenocarcinoma (HR 0.9, 95%CI 0.5–1.6) when analyzed separately^29^.

Lazzati et al. showed that the incidence of esophagogastric cancer in patients undergoing bariatric surgery is statistically lower when compared with a population that did not undergo the procedure. The incidence of esophageal-gastric cancer fell from 6.9 to 4.9 per 100,000 population per year, with a reduction in esophageal cancer from 2.3 to 1.5 and in gastric cancer from 4.6 to 3.3 per 100,000 population per year^38^.

The association between upper digestive tract cancers after bariatric surgery remains controversial, as several procedures can increase the incidence of pre-neoplastic conditions in the esophagus^58^. A French national study evaluated the incidence of colorectal cancer by comparing nearly 2 million individuals who did not undergo bariatric surgery with more than 100,000 who did. The incidence of colorectal cancer has fallen by half in the patients subjected to bariatric surgery^5^.

In the Surgical Procedures and Long-term Effectiveness in Neoplastic Disease Incidence and Death (SPLENDID) matched cohort study, adult patients with a BMI of 35 or greater who underwent bariatric surgery at a U.S. health system between 2004 and 2017 were included^73^. Patients who underwent bariatric surgery were matched in a 1:5 ratio with patients who did not undergo surgery for their obesity, resulting in a total of 30,318 patients. Bariatric surgery (n=5053) included Roux-en-Y gastric bypass (RYGB) and sleeve gastrectomy (SG). During follow-up, 96 patients in the bariatric surgery group and 780 patients in the nonsurgical control group had an incident obesity-associated cancer (incidence rate of 3.0 events *vs*. 4.6 events, respectively, per 1,000 person-years). The cumulative incidence of the primary endpoint at 10 years was 2.9% (95%CI 2.2–3.6%) in the bariatric surgery group and 4.9% (95%CI 4.5–5.3%) in the nonsurgical control group (absolute risk difference, 2.0% [95%CI 1.2–2.7%]; adjusted HR 0.68 [95%CI 0.53–0.87], p=0.002, p<0.05). Cancer-related mortality occurred in 21 patients in the bariatric surgery group and 205 patients in the nonsurgical control group (incidence rate of 0.6 events *vs*. 1.2 events, respectively, per 1,000 person-years). The cumulative incidence of cancer-related mortality at 10 years was 0.8% (95%CI 0.4–1.2%) in the bariatric surgery group and 1.4% (95%CI 1.1–1.6%) in the nonsurgical control group (absolute risk difference, 0.6% [95%CI 0.1–1.0%]; adjusted HR 0.52 [95%CI 0.31–0.88], p=0.01, p<0.05). The authors concluded that, among adults with obesity, bariatric surgery compared with no surgery was associated with a significantly lower incidence of obesity-associated cancer and cancer-related mortality^2^.

### Does the bariatric surgery technique — Roux-en-Y gastric bypass, sleeve gastrectomy, or single anastomosis mini gastric bypass — influence the risk of cancer after the operation?

Bariatric surgery has become a widely performed procedure globally, with approximately 1 million surgeries estimated to occur each year. Among the procedures performed, SG and RYGB account for approximately 80% of surgeries performed in the U.S. Mini gastric bypass (OAGB) is another technique that has been gaining a lot of attention and has a growing number of followers, becoming the most performed procedure in some countries^17,43^.

These three procedures raise some concerns regarding potential carcinogenic characteristics. Specifically, SG is associated with a higher incidence of reflux, esophagitis, and Barrett’s esophagus. RYGB raises concerns due to the exclusion of the stomach, while OAGB is linked to alkaline reflux. The major concern among these issues would be the development of esophageal cancer in this population^47^.

### Sleeve gastrectomy

The incidence of cancer after SG has been a subject of investigation in several studies. According to a systematic review and meta-analysis, bariatric surgery, including SG, is associated with a significant reduction in overall cancer incidence compared to nonsurgical treatment. Specifically, the odds ratio (OR) for cancer incidence in patients undergoing SG was 0.44 (95%CI 0.27–0.70)^14^.

However, there are specific concerns regarding the risk of esophageal cancer post SG due to the potential for increased gastroesophageal reflux disease (GERD) and consequently esophagitis and risk of Barrett’s esophagus. One study found that the crude incidence rate of esophageal cancer in patients undergoing reflux-prone procedures like SG was higher than in nonsurgical controls, but this difference was not significant after adjusting for confounders^3^.

Some case studies reported an incidence of approximately 18% for Barrett’s esophagus, 52% for the need for stomach-protective medication, and 41% for esophagitis within 5 years of evolution^58^. The shape of the gastric tube appears to influence the incidence of reflux, especially when it is made in a twisted manner^26^. However, the incidence of Barrett’s in patients undergoing SG varies widely in the literature. Dantas et al., in an endoscopic evaluation with more than 5 years of follow-up, did not identify any cases of Barrett’s esophagus in their case study^18^. In summary, while sleeve gastrectomy is associated with a reduced overall cancer incidence, there is a notable concern for esophageal cancer due to increased GERD and Barrett’s esophagus. Therefore, SG is generally lower for most cancer types, vigilance for esophageal cancer remains necessary.

### Roux-en-Y gastric bypass

The creation of an excluded gastric chamber in this type of surgery raises concerns mainly due to the difficulty in accessing the excluded stomach. Some studies, in which the excluded chamber was accessed through double-balloon endoscopy, showed an incidence of atrophic gastritis of 14.3%, metaplasia in 11.4%, and *H. pylori* presence in 20% of cases. In addition to the changes mentioned, there appear to be changes in the microenvironment that may predispose to oncogenic molecular mutations^48,52,53^.

The incidence of gastric cancer after RYGB is rare but has been documented in the medical literature. According to a systematic review by Chemaly et al., the occurrence of gastroesophageal cancer post-RYGB is primarily reported through case studies, with 27 out of 44 cases of gastroesophageal cancer occurring in the gastric tube after RYGB^13^. Another systematic review by Dong et al. identified 21 cases of remnant gastric cancer after RYGB, with a median time to diagnosis of 11 years postoperatively^20^. Additionally, Doukas et al.^21^ reported an increasing trend of gastric cancer cases in the excluded stomach post-RYGB, with 77% of these cancers diagnosed at an advanced stage^13,20,21^.

In summary, while the exact incidence rate is not well-defined due to the rarity and the nature of case reports, available data suggest that gastric cancer can occur in the remnant stomach or gastric tube after RYGB, typically many years postoperatively. Although rare, this warrants awareness and long-term surveillance in high-risk patients.

### Mini gastric bypass

Despite being a relatively recent procedure in the arsenal of surgical treatment for obesity, OAGB brings with it a problem already faced in gastrectomy surgery with B-II reconstruction, which is alkaline reflux. Alkaline reflux can cause changes in the gastric mucosa and esophageal mucosa, with some studies reporting its presence in 21% of cases, potentially increasing cancer incidence^35^. Gastroesophageal cancer appears to be the most commonly reported type of cancer following mini gastric bypass (OAGB), although the incidence is not significantly higher compared to RYGB. According to a systematic review and meta-analysis by Chemaly et al., 37.5% of gastroesophageal cancers after OAGB were located in the gastric tube, compared to 61% after RYGB, with an odds ratio of 0.38, indicating no significant increase in cancer occurrence in the gastric tube after OAGB compared to RYGB^13^.

The arguments that bile reaches the terminal ileum diluted are unfounded, since physiology studies show that 90% of bile reaches that location intact, where it is later reabsorbed. This technique should be used with great caution, since the potential harmful effects, such as the development of cancer in the gastric remnant and esophagus, may only become apparent 30 to 40 years postoperatively^1,6,19,35^.

In conclusion, the incidence of cancer in patients undergoing bariatric surgery is low. Large systematic reviews and meta-analyses list a small number of cases relative to the total number of surgeries performed worldwide, demonstrating that the benefits of surgery, in terms of cancer incidence reduction, outweigh the risks, regardless of the surgical technique employed^43,44,45^. Therefore, while the incidence of cancer in patients undergoing bariatric surgery remains numerically low, it is a concern, and surgeons must remain vigilant to avoid understanding symptoms and delaying diagnosis. On the other hand, there is sufficient evidence to suggest that bariatric surgery reduces the incidence of many types of cancer. However, indications for surgery should be thoroughly discussed with multidisciplinary teams, avoiding exaggeration and sensationalism, especially regarding procedures that increase the incidence of gastroesophageal reflux.

## References

[1] Ahn HS, Kim JW, Yoo MW, Park DJ, Lee HJ, Lee KU (2008). Clinicopathological features and surgical outcomes of patients with remnant gastric cancer after a distal gastrectomy. Ann Surg Oncol.

[2] Aminian A, Wilson R, Al-Kurd A, Tu C, Milinovich A, Kroh M (2022). Association of bariatric surgery with cancer risk and mortality in adults with obesity. JAMA.

[3] Andalib A, Bouchard P, Demyttenaere S, Ferri LE, Court O (2021). Esophageal cancer after sleeve gastrectomy: a population-based comparative cohort study. Surg Obes Relat Dis.

[4] Arnold M, Pandeya N, Byrnes G, Renehan PAG, Stevens GA, Ezzati PM (2015). Global burden of cancer attributable to high body-mass index in 2012: a population-based study. Lancet Oncol.

[5] Bailly L, Fabre R, Pradier C, Iannelli A (2020). Colorectal cancer risk following bariatric surgery in a nationwide study of french individuals with obesity. JAMA Surg.

[6] Basso L, Gallo G, Biacchi D, Carati MV, Cavallaro G, Esposito l (2022). Role of new anatomy, biliopancreatic reflux, and helicobacter pylori status in postgastrectomy stump cancer. J Clin Med.

[7] Bhaskaran K, Douglas I, Forbes H, dos-Santos-Silva I, Leon DA, Smeeth L (2014). Body-mass index and risk of 22 specific cancers: a population-based cohort study of 5·24 million UK adults. Lancet.

[8] Brenner DR, Ruan Y, Shaw E, O’Sullivan D, Poirier AE, Heer E (2019). Age-standardized cancer-incidence trends in Canada, 1971-2015. CMAJ.

[9] Brown KF, Rumgay H, Dunlop C, Ryan M, Quartly F, Cox A (2018). The fraction of cancer attributable to modifiable risk factors in England, Wales, Scotland, Northern Ireland, and the United Kingdom in 2015. Br J Cancer.

[10] Calle EE, Kaaks R (2004). Overweight, obesity and cancer: epidemiological evidence and proposed mechanisms. Nat Rev Cancer.

[11] Calle EE, Rodriguez C, Walker-Thurmond K, Thun MJ (2003). Overweight, obesity, and mortality from cancer in a prospectively studied cohort of U.S. adults. N Engl J Med.

[12] Castagneto-Gissey L, Casella-Mariolo J, Casella G, Mingrone G (2020). Obesity surgery and cancer: what are the unanswered questions?. Front Endocrinol (Lausanne).

[13] Chemaly R, Diab S, Khazen G, Al-Hajj G (2022). Gastroesophageal cancer after gastric bypass surgeries: a systematic review and meta-analysis. Obes Surg.

[14] Chen ZW, Jin T, Liang PP, Li ZD, He FJ, Chen ZH (2024). Incidence of cancer for patients after bariatric surgery: evidence from 33 cohort studies. Surg Obes Relat Dis.

[15] Chittajallu V, Mansoor E, Perez J, Omar YA, Firkins SA, Yoo H (2023). Association of bariatric surgery with risk of incident obesity-associated malignancies: a multi-center population-based study. Obes Surg.

[16] Christou NV, Sampalis JS, Liberman M, Look D, Auger S, McLean APH (2004). Surgery decreases long-term mortality, morbidity, and health care use in morbidly obese patients. Ann Surg.

[17] Clapp B, Ponce J, Corbett J, Ghanem OM, Kurian M, Rogers AM (2024). American Society for Metabolic and Bariatric Surgery 2022 estimate of metabolic and bariatric procedures performed in the United States. Surg Obes Relat Dis.

[18] Dantas ACB, Coutinho JL, Meira JD, De Moura DTH, Pajecki D, Santo MA (2024). Lots of reflux, but no Barrett: real-world data on the incidence of gastroesophageal reflux on routine endoscopic follow-up more than 5 years after sleeve gastrectomy. J Gastrointest Surg.

[19] Diogo A, Botelho LF, Nishiyama A, Zumpano LE, Monte RC, Rosa SC (2016). Gastric stump cancer after gastrectomy by gastroduodenal peptic ulcer. Arq Bras Cir Dig.

[20] Dong SL, Liang YL, Wang CC, Dong ZY (2022). Occurrence of gastric cancer after laparoscopic Roux-en-Y gastric bypass: a systematic review. Zhonghua Wei Chang Wai Ke Za Zhi.

[21] Doukas SG, Doukas PG, Vageli DP, Broder A (2023). Gastric cancer after Bariatric Bypass Surgery. Do they relate? (a systematic review). Obes Surg.

[22] Doumouras AG, Lovrics O, Paterson JM, Sutradhar R, Paszat L, Sivapathasundaram B (2023). Residual risk of breast cancer after bariatric surgery. JAMA Surg.

[23] Ebrahimi R, Kermansaravi M, Khalaj A, Eghbali F, Mousavi A, Pazouki A (2019). Gastro-intestinal tract cancers following bariatric surgery: a narrative review. Obes Surg.

[24] Estivaleti JM, Guzman-Habinger J, Lobos J, Azeredo CM, Claro R, Ferrari G (2022). Time trends and projected obesity epidemic in Brazilian adults between 2006 and 2030. Sci Rep.

[25] Feigelson HS, Bodelon C, Powers JD, Curtis RE, Buist DSM, Veiga LHS (2021). Body mass index and risk of second cancer among women with breast cancer. J Natl Cancer Inst.

[26] Ferraz AAB, Silva JTD, Santa-Cruz F, Aquino MAR, Siqueira LT, Kreimer F (2020). The impact of the gastric twist on esophagitis progression after sleeve gastrectomy: mid-term endoscopic findings. Obes Surg.

[27] Freitas WR, Oliveira LVF, Perez EA, Ilias EJ, Lottenberg CP, Silva AS (2018). Systemic inflammation in severe obese patients undergoing surgery for obesity and weight-related diseases. Obes Surg.

[28] Hampl SE, Hassink SG, Skinner AC, Armstrong SC, Barlow SE, Bolling CF (2023). Clinical practice guideline for the evaluation and treatment of children and adolescents with obesity. Pediatrics.

[29] Åkerström JH, Santoni G, von Euler Chelpin M, Chidambaram S, Markar SR, Maret-Ouda J (2023). Decreased risk of esophageal adenocarcinoma after gastric bypass surgery in a cohort study from 3 nordic countries. Ann Surg.

[30] Hopkins BD, Goncalves MD, Cantley LC (2016). Obesity and cancer mechanisms: cancer metabolism. J Clin Oncol.

[31] Islami F, Sauer AG, Gapstur SM, Jemal A (2019). Proportion of cancer cases attributable to excess body weight by US state, 2011-2015. JAMA Oncol.

[32] Iyengar NM, Gucalp A, Dannenberg AJ, Hudis CA (2016). Obesity and cancer mechanisms: tumor microenvironment and inflammation. J Clin Oncol.

[33] Kaidar-Person O, Bar-Sela G, Person B (2011). The two major epidemics of the twenty-first century: obesity and cancer. Obes Surg.

[34] Kodaira K, Abe FC, Galvão TF, Silva MT (2021). Time-trend in excess weight in Brazilian adults: a systematic review and meta-analysis. PLoS One.

[35] Kondo K, Akiyama S, Ito K, Yokoyama Y, Takagi H, Takahashi T (1993). Recent advances in management of digestive cancers.

[36] Kristensson FM, Andersson-Assarsson JC, Peltonen M, Jacobson P, Ahlin S, Svensson PA (2024). Breast cancer risk after bariatric surgery and influence of insulin levels: a nonrandomized controlled trial. JAMA Surg.

[37] Lauby-Secretan B, Scoccianti C, Loomis D, Grosse Y, Bianchini F, Straif K (2016). Body fatness and cancer--viewpoint of the IARC Working Group. N Engl J Med.

[38] Lazzati A, Poghosyan T, Touati M, Collet D, Gronnier C (2023). Risk of esophageal and gastric cancer after bariatric surgery. JAMA Surg.

[39] Luo J, Hendryx M, Manson JE, Figueiredo JC, LeBlanc ES, Barrington W (2019). Intentional weight loss and obesity-related cancer risk. JNCI Cancer Spectr.

[40] Mackenzie H, Markar SR, Askari A, Faiz O, Hull M, Purkayastha S (2018). Obesity surgery and risk of cancer. Br J Surg.

[41] Center for Disease Control and Prevention (2019). Obesity and cancer.

[42] Nielsen J, Narayan KV, Cunningham SA (2023). Incidence of obesity across adulthood in the United States, 2001-2017-a national prospective analysis. Am J Clin Nutr.

[43] Onzi TR, Salgado W, Bastos ELS, Dantas ACB, Silva LB, Oliveira AA (2024). Efficacy and safety of one anastomosis gastric bypass in surgical treatment of obesity: systematic review and meta-analysis of randomized controlled trials. Arq Bras Cir Dig.

[44] Parmar C, Pouwels S (2022). Oesophageal and gastric cancer after bariatric surgery: an up-to-date systematic scoping review of literature of 324 cases. Obes Surg.

[45] Parmar C, Zakeri R, Abouelazayem M, Shin TH, Aminian A, Mahmoud T (2022). Esophageal and gastric malignancies after bariatric surgery: a retrospective global study. Surg Obes Relat Dis.

[46] Pati S, Irfan W, Jameel A, Ahmed S, Shahid RK (2023). Obesity and cancer: a current overview of epidemiology, pathogenesis, outcomes, and management. Cancers (Basel).

[47] Plat VD, Kasteleijn A, Greve JWM, Luyer MDP, Gisbertz SS, Demirkiran A (2021). Esophageal cancer after bariatric surgery: increasing prevalence and treatment strategies. Obes Surg.

[48] Ravacci GR, Ishida R, Torrinhas RS, Sala P, Machado NM, Fonseca DC (2019). Potential premalignant status of gastric portion excluded after Roux en-Y gastric bypass in obese women: a pilot study. Sci Rep.

[49] Recalde M, Pistillo A, Davila-Batista V, Leitzmann M, Romieu I, Viallon V (2023). Longitudinal body mass index and cancer risk: a cohort study of 2.6 million Catalan adults. Nat Commun.

[50] Renehan AG, Zwahlen M, Egger M (2015). Adiposity and cancer risk: new mechanistic insights from epidemiology. Nat Rev Cancer.

[51] Rock CL, Thomson C, Gansler T, Gapstur SM, McCullough ML, Patel AV (2020). American Cancer Society guideline for diet and physical activity for cancer prevention. CA Cancer J Clin.

[52] Safatle-Ribeiro A, Kuga R, Iriya K, Ribeiro U, Faintuch J, Ishida RK (2007). What to expect in the excluded stomach mucosa after vertical banded Roux-en-Y gastric bypass for morbid obesity. J Gastrointest Surg.

[53] Safatle-Ribeiro AV, Ribeiro U (2023). Is gastric cancer after bariatric surgery on the rise? Will history repeat itself?. Chin J Cancer Res.

[54] Schauer DP, Feigelson HS, Koebnick C, Caan B, Weinmann S, Leonard AC (2019). Bariatric surgery and the risk of cancer in a large multisite cohort. Ann Surg.

[55] Schauer DP, Feigelson HS, Koebnick C, Caan B, Weinmann S, Leonard AC (2017). Association between weight loss and the risk of cancer after bariatric surgery. Obesity (Silver Spring).

[56] Schiel WA, Peppe AP, Weiss AG, Cortiano LGG, Branco AJ, Almeida FE (2023). Laparoscopic and laparotomy bariatric surgery in a public hospital in Brazil: are there differences in costs and complications?. Arq Bras Cir Dig.

[57] Schmitz KH, Neuhouser ML, Agurs-Collins T, Zanetti KA, Cadmus-Bertram L, Dean LT (2013). Impact of obesity on cancer survivorship and the potential relevance of race and ethnicity. J Natl Cancer Inst.

[58] Sebastianelli L, Benois M, Vanbiervliet G, Bailly L, Robert M, Turrin N (2019). Systematic endoscopy 5 years after sleeve gastrectomy results in a high rate of Barrett’s esophagus: results of a multicenter study. Obes Surg.

[59] Singh A, Mayengbam SS, Yaduvanshi H, Wani MR, Bhat MK (2022). Obesity programs macrophages to support cancer progression. Cancer Res.

[60] Siqueira LT, Wanderley MSO, Silva RA, Pereira ASA, Lima JL, Ferraz AAB (2018). A screening study of potential carcinogen biomarkers after surgical treatment of obesity. Obes Surg.

[61] Sjöström L, Narbro K, Sjöström CD, Karason K, Larsson B, Wedel H (2007). Effects of bariatric surgery on mortality in Swedish obese subjects. N Engl J Med.

[62] Smith CJ, Perfetti TA, Hayes AW, Berry SC (2020). Obesity as a source of endogenous compounds associated with chronic disease: a review. Toxicol Sci.

[63] Song M, Giovannucci E (2016). Preventable incidence and mortality of carcinoma associated with lifestyle factors among white adults in the United States. JAMA Oncol.

[64] Stierman B, Afful J, Carroll MD, Chen TC, Davy O, Fink S (2021). National Health and Nutrition Examination Survey 2017–March 2020 prepandemic data files-development of files and prevalence estimates for selected health outcomes. Natl Health Stat Report.

[65] Stroud AM, Dewey EN, Husain FA, Fischer JM, Courcoulas AP, Flum DR (2020). Association between weight loss and serum biomarkers with risk of incident cancer in the Longitudinal Assessment of Bariatric Surgery cohort. Surg Obes Relat Dis.

[66] Sung H, Hyun N, Leach CR, Yabroff KR, Jemal A (2020). Association of first primary cancer with risk of subsequent primary cancer among survivors of adult-onset cancers in the United States. JAMA.

[67] Sung H, Siegel RL, Rosenberg PS, Jemal A (2019). Emerging cancer trends among young adults in the USA: analysis of a population-based cancer registry. Lancet Public Health.

[68] Tee MC, Cao Y, Warnock GL, Hu FB, Chavarro JE (2013). Effect of bariatric surgery on oncologic outcomes: a systematic review and meta-analysis. Surg Endosc.

[69] Trust for America’s Health (2023). State of obesity 2023: better policies for a healthier America. Trust for America’s Health.

[70] Tsui ST, Yang J, Zhang X, Docimo S, Spaniolas K, Talamini MA (2020). Development of cancer after bariatric surgery. Surg Obes Relat Dis.

[71] Valezi AC, Campos ACL, Von Bahten LC (2023). Brazilian multi-society position statement on emerging bariatric and metabolic surgical procedures. Arq Bras Cir Dig.

[72] Ward ZJ, Bleich SN, Cradock AL, Barrett JL, Giles CM, Flax C (2019). Projected U.S. state-level prevalence of adult obesity and severe obesity. N Engl J Med.

[73] Wilson R, Aminian A (2023). Obesity-associated cancer risk reduction after metabolic surgery: insights from the SPLENDID study and the path forward. Surg Obes Relat Dis.

[74] Wilson RB, Lathigara D, Kaushal D (2023). Systematic review and meta-analysis of the impact of bariatric surgery on future cancer risk. Int J Mol Sci.

